# Aberrant RNA Splicing Events Driven by Mutations of RNA-Binding Proteins as Indicators for Skin Cutaneous Melanoma Prognosis

**DOI:** 10.3389/fonc.2020.568469

**Published:** 2020-10-15

**Authors:** Chao Mei, Pei-Yuan Song, Wei Zhang, Hong-Hao Zhou, Xi Li, Zhao-Qian Liu

**Affiliations:** ^1^Department of Clinical Pharmacology, Hunan Key Laboratory of Pharmacogenetics, Key Laboratory of Biological Nanotechnology of National Health Commission, and National Clinical Research Center for Geriatric Disorders, Xiangya Hospital, Central South University, Changsha, China; ^2^Engineering Research Center for Applied Technology of Pharmacogenomics of Ministry of Education, Institute of Clinical Pharmacology, Central South University, Changsha, China

**Keywords:** skin cutaneous melanoma, RNA-binding protein, mutation, alternative splicing, prognosis

## Abstract

The worldwide incidence of skin cutaneous melanoma (SKCM) is increasing at a more rapid rate than other tumors. Aberrant alternative splicing (AS) is found to be common in cancer; however, how this process contributes to cancer prognosis still remains largely unknown. Mutations in RNA-binding proteins (RBPs) may trigger great changes in the splicing process. In this study, we comprehensively analyzed DNA and RNA sequencing data and clinical information of SKCM patients, together with widespread changes in splicing patterns induced by RBP mutations. We screened mRNA expression-related and prognosis-related mutations in RBPs and investigated the potential affections of RBP mutations on splicing patterns. Mutations in 853 RBPs were demonstrated to be correlated with splicing aberrations (*p* < 0.01). Functional enrichment analysis revealed that these alternative splicing events (ASEs) may participate in tumor progress by regulating the modification process, cell-cycle checkpoint, metabolic pathways, MAPK signaling, PI3K-Akt signaling, and other important pathways in cancer. We also constructed a prediction model based on overall survival-related AS events (OS-ASEs) affected by RBP mutations, which exhibited a good predict efficiency with the area under the curve of 0.989. Our work highlights the importance of RBP mutations in splicing alterations and provides effective biomarkers for prediction of prognosis of SKCM.

## Introduction

Skin cutaneous melanoma (SKCM) is the deadliest type of skin cancer, comprising <5% of skin cancers but accounting for the majority of skin malignancy-induced deaths (1, 2). SKCM patient survival depends largely on early detection and diagnosis. Assessment and stratification of patients ahead of clinical therapy aid in identifying individuals with high risk of recurrence or poor survival outcome and may help to inform clinical decisions or potential targeted therapy strategies.

Both genetic and epigenetic alterations that interfere with normal cell physiological function are the fundamental reason of tumorigenesis (3–7). Alterations in transcriptome provide one of the biggest possibilities of proteome and molecular diversity of human cells. Alternative splicing (AS) is a posttranscriptional regulatory mechanism that transcribes a single form of pre-mRNA into multiple mature RNA molecules with different sequences and structures, which exist in almost 95% genes in human genome and provide large potential for multiple pathologies including cancer (8–11). Splicing is highly altered in tumor (12, 13). AS aberrations may lead to transcriptome variations in some cancer-related genes and in turn impact tumor progression as well as therapy resistance (14–17). These findings result in a growing interest in studying the role of aberrant AS in tumor progression.

ASEs have exhibited great potential to act as a biomarker for prognosis prediction in several tumor types. Therefore, it would be desirable to have a better understanding of the AS events (ASEs) in SKCM, which would provide potential for the development of novel clinical biomarkers as well as assist in more effective therapeutic decisions (13, 18). Importantly, AS is regulated by a number of RNA-binding proteins (RBPs), mutations of which may trigger widespread splicing aberrations in downstream spliced genes (19–21). RBPs are a category of proteins with unique RNA-binding domains and are usually dysregulated in tumors, which allow them to regulate a large array of transcript processes (22–26).

It is now clear that either mutations or expression changes in RBPs along with corresponding AS aberrations potentially have great value in exploring tumorigenesis and progression. Mutations or expression changes in splicing regulatory factors have been described as oncogenic drivers (27, 28) and would contribute to affecting oncogenic processes (29–31). However, recent studies focused on SKCM have several limitations: they only investigate one or a few genes, they lack effective biomarker identification, they are only reliable to a specific patient specimen, and there is no comprehensive investigation on how splicing regulatory factor aberrations contribute to SKCM development (32–34). There still exists a demand of systematic investigation on how mutations or expression patterns of RBPs along with related AS changes contribute to SKCM survival outcomes. Here, we systematically investigated the DNA and RNA sequencing data as well as clinical information of SKCM samples obtained from the Cancer Genome Atlas (TCGA) dataset, combined with ASE information from TCGA SpliceSeq data, so as to comprehensively describe the complex network of alterations in RBPs and their global effects in ASEs.

## Materials and Methods

### Data Collection

Alternative splicing data of 103 SKCM patients were downloaded from TCGA SpliceSeq (https://bioinformatics.mdanderson.org/TCGASpliceSeq), a resource for investigating AS patterns in 33 types of human tumor (35). The Percent Spliced In (PSI) value, which ranges from zero to one, was commonly used to quantify ASEs. ASEs with PSI values >75% were selected in this study. Missing values were supplied using “impute” package in R (36–38). ASEs with PSI average < 0.05 or standard deviation < 0.01 were removed. ASE was named as the combination of gene symbol, splicing type, and splicing ID number. DNA-seq data, RNA-seq data, and clinical information were obtained from the TCGA data portal (https://portal.gdc.cancer.gov/repository). Samples with follow-up time of <90 days were removed from the following study. Information of 1350 RBPs was obtained from a previous study (Supplementary Table 1) (39).

### Alternative Splicing Events

After processing the alternative splicing data according to the method described above, a total of 26,919 ASEs in 103 SKCM samples were included in our study. There are seven types of alternative splicing events (ASE), including 2,332 alternate acceptor site events (AA), 2,056 alternate donor site events (AD), 5,126 alternate promoter events (AP), 5,096 alternate terminator events (AT), 1,0304 exon skip events (ES), 103 mutually exclusive exons events (ME), and 1,902 retained intron events (RI). The Percent Spliced In (PSI) value, a common value for quantifying ASEs, ranged from zero to one.

### Mutation Analysis

Samples with both RBP mutation data and AS information were selected in the study. The frequencies of somatic mutations were calculated for each RBPs, and the correlation between mutations and PSI value of ASEs was tested using the Wilcoxon–Mann–Whitney test (*p* < 0.01). The waterfall plots were built using the “GenomeInfoDbData” and “GenVisR” package in R. The balloon plot was visualized using the “ggpubr” and “ggplot2” package. The Upset plot was constructed with the “UpSetR” package in R.

### RBP Mutations and Differential Expression

Differential expression was analyzed between samples with or without RBP mutations. The Wilcoxon–Mann–Whitney test was applied for estimating the correlations between RBP mutations and mRNA expression alterations, and RBPs were considered differentially expressed if *p*-value < 0.05. The association between RBPs with mRNA expression-related mutations and PSI value of ASEs was evaluated using Pearson correlation analysis; outcomes with |Cor|>0.5 and *p* < 0.05 were regarded as significant correlation. The bubble plots were visualized using “ggplot2” package in R.

### RBP Mutations and Prognosis Analysis

The association between RBP mutations and prognosis of SKCM were investigated using Kaplan–Meier survival estimate analysis. A total of 177 samples with both clinical information and DNA sequencing data were selected in study. The analyses were realized using “survival” package in R, and significant correlations were identified with *p* < 0.05.

### Protein–Protein Interaction Network and Functional Enrichment Analysis

The spliced genes of most significant ASEs (*p* < 1.00E-05) were input to the String database (https://string-db.org/), and the highest confidence (0.9) was chosen for confirming the interactions. The protein–protein interaction (PPI) network was visualized using Cytoscape. Gene Ontology (GO) analysis was investigated using the “clusterProfiler” package in R, and Kyoto Encyclopedia of Genes and Genomes (KEGG) enrichment analysis was performed in KOBAs (40), with a criterion of adjusted *P*-value of < 0.05. The results are shown in bar plots.

### Identification of OS-ASEs

Survival information of SKCM patients was obtained from TCGA. Univariable Cox regression analysis was performed to evaluate the prognostic value of every ASE, and events with *p*-value < 0.05 were identified as OS-ASEs. The Upset plot was constructed to visualize the quantitative analysis of the intersections of seven types of ASEs. A volcano plot showed the overview of survival-related and unrelated ASEs. The most significant OS-ASEs of each type of ASEs are displayed in bubble plots described above. Pearson correlation analysis was used to test the correlation between RBP expression and PSIs of OS-ASEs; the correlation network was built based on the outcomes with |Cor|>0.6 and *p* < 0.05.

### Construction of Prediction Model Based on OS-ASEs

OS-ASEs were firstly screened by the least absolute shrinkage and selection operator (LASSO) regression analysis, and then multivariate Cox regression analysis was performed to screen the significant prognostic OS-ASEs and build the prediction model. The area under the curve (AUC) value under the receiver operator characteristic (ROC) curve was calculated using the “survivalROC” package in R, which was used to evaluate the prognosis predictive efficiencies. Samples were then divided into high-risk and low-risk groups according to the median risk score calculated by our prediction model. Kaplan–Meier survival analyses were used for evaluating the prognostic efficiency of different prognostic indexes (PIs) in high- and low-risk patients. The analysis was proceeded with the “survival” package, and survival curves were plotted using the “survminer” package in R. Heatmap was visualized using the “pheatmap” package. Univariate Cox regression analysis and multivariate Cox regression analysis were applied to evaluate the prognosis value of risk factors including risk score quartiles, age, gender, tumor stage, and TMN stage in overall survival of SKCM patients. Factors with HR>1 or <1 were identified as high- and low-risk factors (*p* < 0.05), respectively.

## Results

### Splicing Alterations Driven by RBP Mutations in SKCM

**Figure 1:**
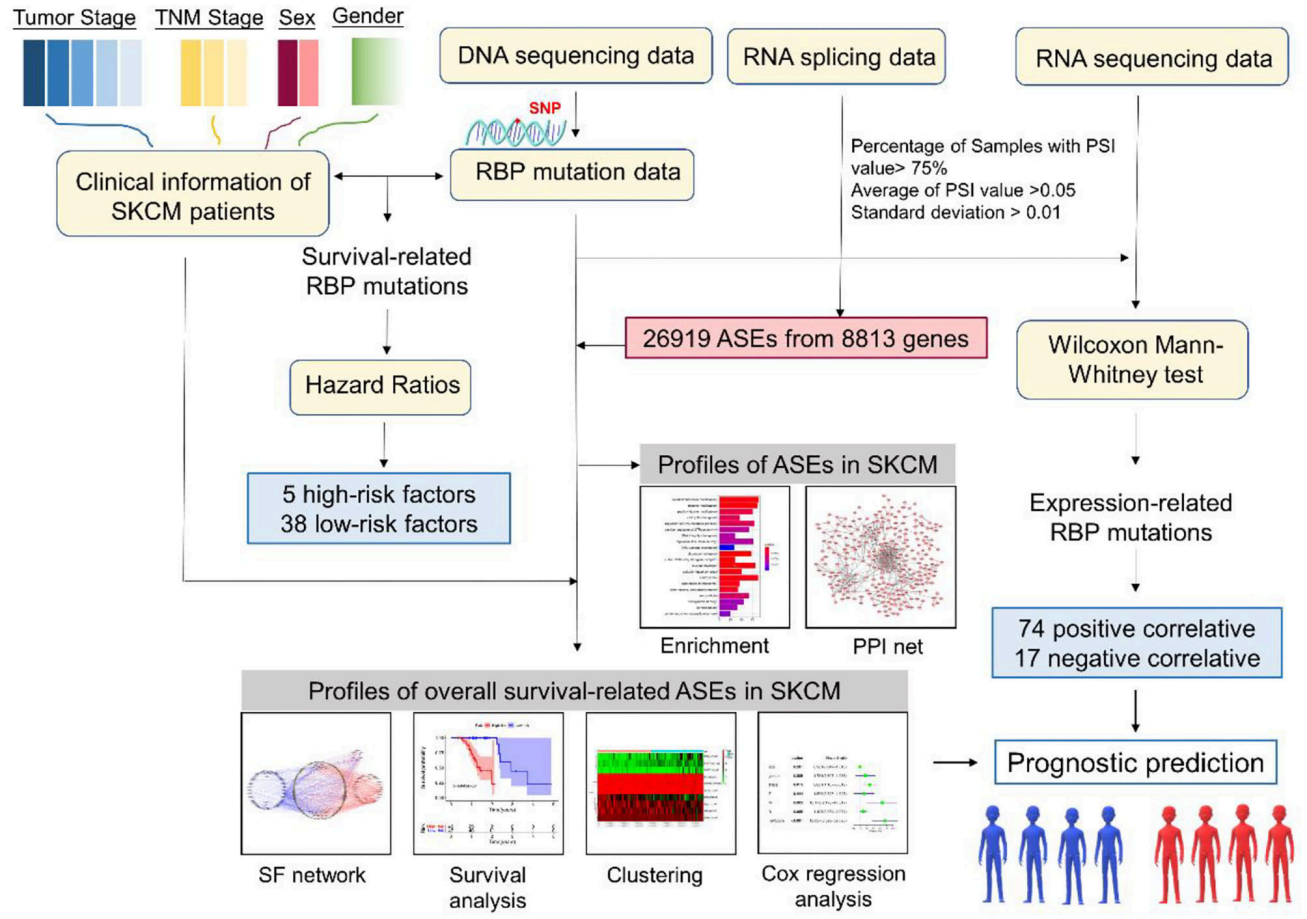
Flow diagram of the research methodology. RBP, RNA-binding protein; ASE, alternative splicing event; SF, splicing factor; PPI, protein–protein interaction.

**Figure 2:**
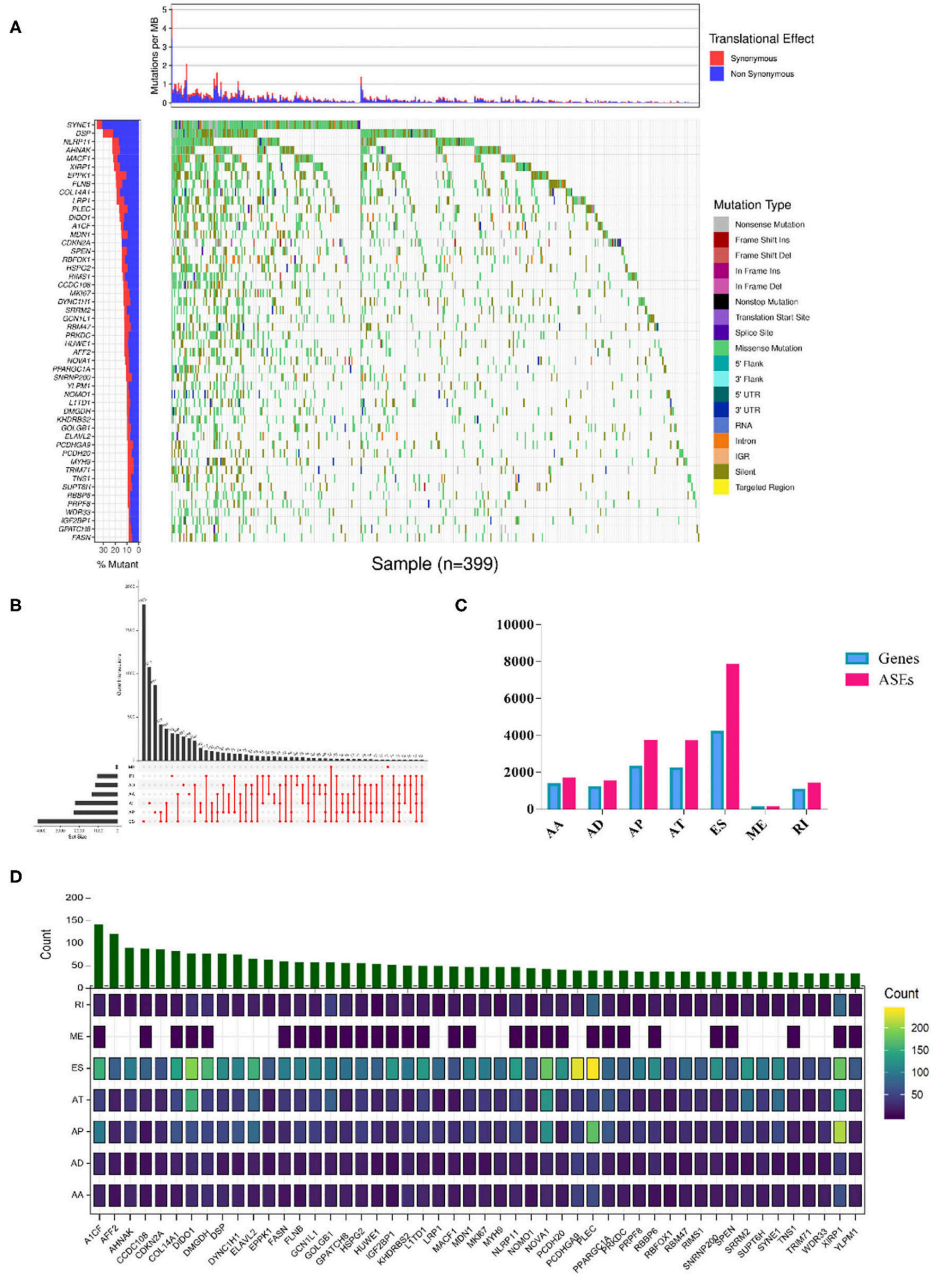
RBP mutations triggered splicing event alterations. **(A)** Among the 853 RBPs whose mutations showed significant correlations with aberrant ASEs (Wilcoxon test *P*-values < 0.01). The mutation situations of the top 50 RBPs with the highest mutant frequency were exhibited. The y-axis represents each RBP and sorted by mutation frequency. The x-axis stands for different SKCM samples. The color indicates different mutation types. The bar plot above represents the mutation frequency with synonymous (red) and non-synonymous (blue) translational effects for each SKCM patient. **(B)** Upset plot of each splicing pattern affected by RBP mutations. The black bar on the left side indicates the event number of specific splicing type, and the red dots on the right side represent the intersections of ASEs. **(C)** Distribution of seven types of ASEs and their corresponding genes. The x-axis represents each kind of ASEs (AA, AD, AP, AT, ES, ME, RI), and the y-axis represents the number of ASEs (red) or the corresponding spliced genes (blue). **(D)** Overview of splicing events per RBP with the highest mutation frequency. The color intensity indicates the occurrence frequency of each splicing pattern, and the above histogram represented the counts of ASEs affected by corresponding RBPs.

The overall analysis process is presented in Figure 1. We first systematically investigated the splicing process alterations induced by RBP mutations. DNA sequence data of 467 SKCM patients were downloaded from TCGA, while ASE information of 103 SKCM samples was obtained from TCGA SpliceSeq data. The mutant situations of 1,350 known and predicted RBPs were studied in 467 SKCM samples, 452 of which were found to carry mutations in RBPs. Mutant situations of the top 50 RBPs with the highest mutation frequency were exhibited using a waterfall plot (Figure 2A). The mutant information was then combined with the Percent Spliced In (PSI) value of ASEs and applied to the Wilcoxon–Mann–Whitney test. As a result, mutations in 853 RBPs were found to be obviously correlated with 19748 different ASEs (Supplementary Table 2), including 1,655 AAs in 1,334 genes, 1,478 ADs in 1,161 genes, 3,681 APs in 2,280 genes, 3,665 ATs in 2,206 genes, 7,802 ESs in 4,178 genes, 85 MEs in 84 genes, and 1,382 RIs in 1,042 genes (Figures 2B,C). The top 50 RBPs with the highest mutation frequency and the corresponding ASEs of each splicing pattern are shown in Figure 2D. The above results indicated that RBP mutations were significantly correlated with extensive AS alterations in SKCM.

### RBP Mutation-Induced mRNA Expression Alterations

**Figure 3:**
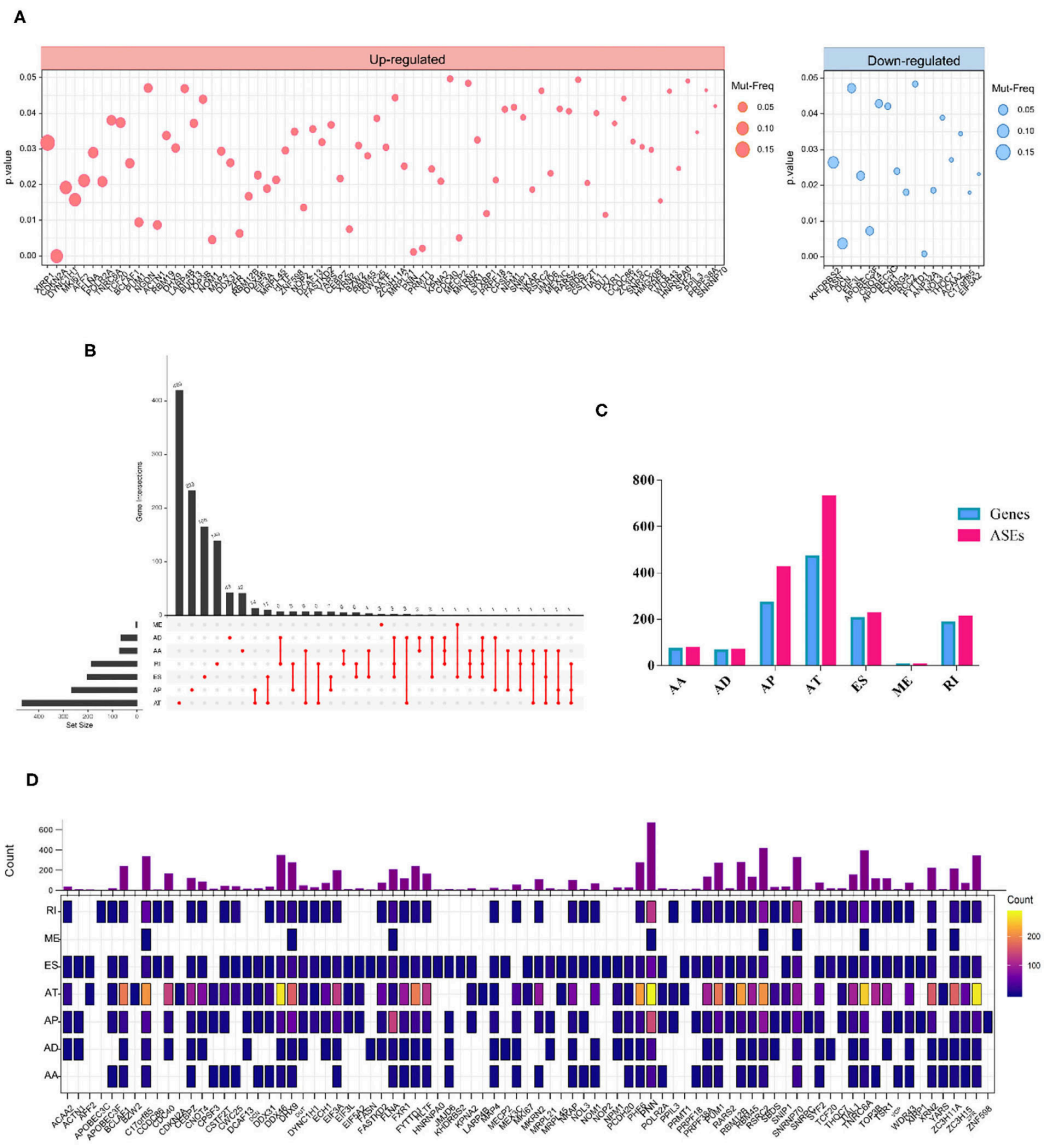
RBP mutations induced mRNA expression alterations**. (A)** A total of 91 RBPs exhibit significant different mRNA expression levels induced by mutations (Wilcoxon test *P*-values < 0.05). The left and right panels indicate the up- and downregulation of the mRNA expression level of tested RBPs. The circle size corresponds to the mutation frequency of SKCM samples. **(B)** Upset plot showed splicing alterations induced by mRNA expression-associated RBP mutations. The black bar on the left represents the counts of different splicing patterns, while the red dots and lines on the right indicate the intersections of ASEs. **(C)** Bar graph represents the number of each splicing type as well as corresponding spliced genes. **(D)** Distribution of splicing events per RBP with mRNA expression-affecting mutations. The color intensity indicates the occurrence counts of each type of ASEs.

We also investigated whether the aberrant splicing process driven by RBP mutations occurred as a consequence of mRNA expression alterations of RBPs. To screen out mutations in RBPs that altered the mRNA expression level, we combined DNA and RNA sequencing data downloaded from TCGA and analyzed. As a result, a total of 91 RBPs exhibited a significant differential expression between wild and mutant genotypes (Supplementary Table 3). In more details, RBPs with upregulated and downregulated mRNA expression levels in mutant samples compared to wild type were exhibited, respectively (Figure 3A). The overview of mutation situations of 91 RBPs in 292 SKCM samples were visualized (Supplementary Figure 1).

Then, we excavated the splicing alterations that may occur as a consequence of expression-associated RBP mutations described above. The association between 91 RBP and PSI values of ASEs was tested in 103 SKCM samples (Supplementary Table 4). Significant associations were found in 1728 ASEs |Cor|>0.5, *p* < 0.05), including 74 AAs in 71 genes, 66 ADs in 64 genes, 422 APs in 270 genes, 728 ATs in 470 genes, 225 ESs in 204 genes, 4 MEs in 4 genes, and 209 RIs in 185 genes (Figures 3B,C). This result indicated that a single gene can undergo multiple types of ASEs. Besides, among seven types of ASEs, the incidence of AT was most frequently followed by AP, while the rarest type was ME events. The detailed regulatory network of each RBPs on different splicing pattern is exhibited in Figure 3D.

Collectively, the above results indicated that RBP genotypes are frequently altered in SKCM tumors and may contribute to affecting the mRNA expression, which was consistent with previous researches focused on other human tumors (39). The expression-associated RBP mutations discovered in this study and their affecting ASEs provide new information about RBP mutations with a potential role in physiological function regulation of SKCM.

### RBP Mutations Associate With Survival Outcome of SKCM Patients

**Figure 4:**
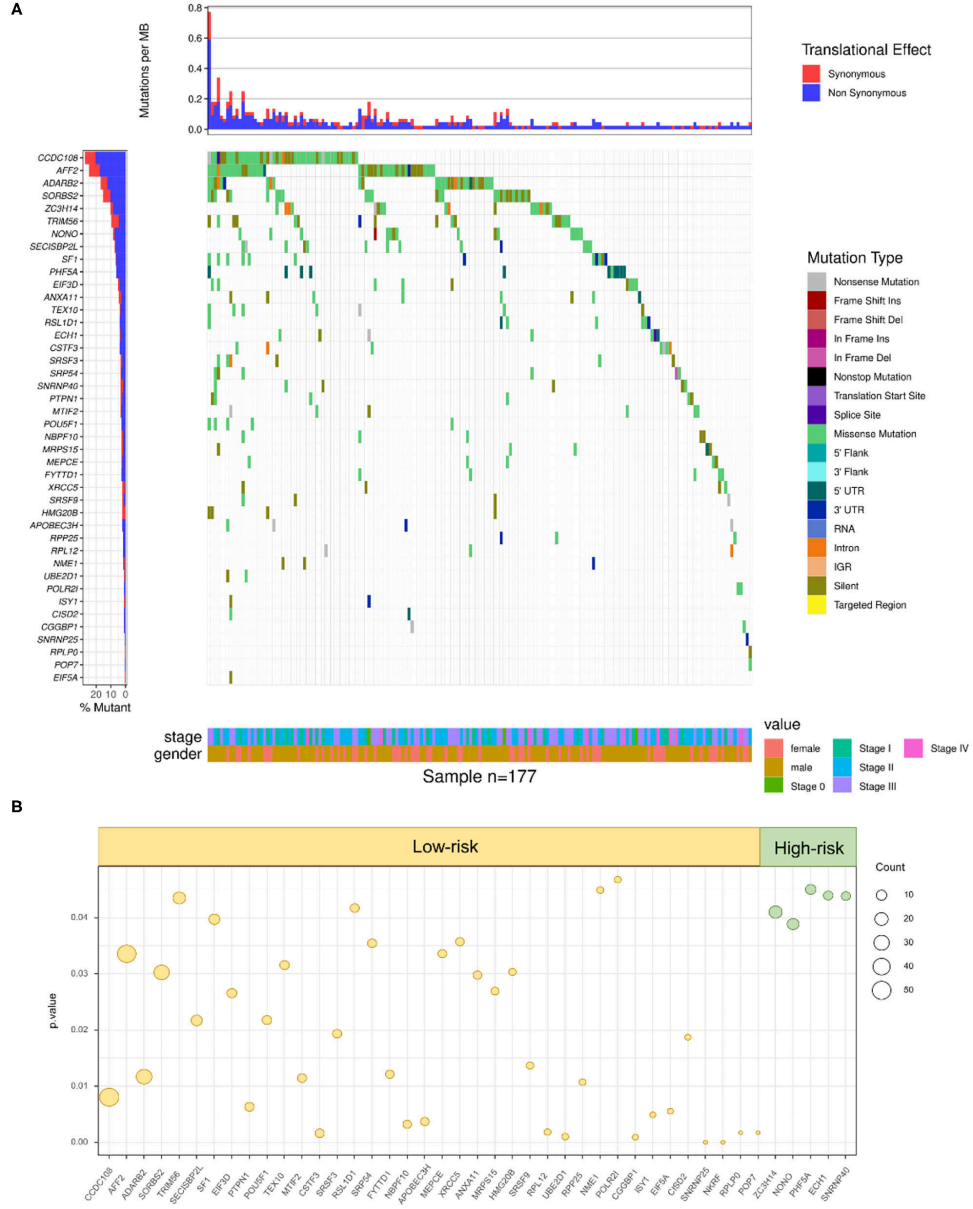
RBP mutations correlated with SKCM prognosis. **(A)** A total of 43 RBPs were demonstrated to carry prognosis-associated mutations (Kruskal–Wallis H test *P*-values < 0.05). The y-axis represents each RBP and sorted by mutation frequency. The x-axis stands for different SKCM samples. The color indicates different mutation type. The bar plot above represents the mutation frequency with synonymous (red) and non-synonymous (blue) translational effects of each sample**. (B)** High-risk factors (right panel) and low-risk factors (left panel) of prognosis-related RBP mutations. The circle size stands for count of samples with mutations.

To further define whether mutations in RBPs could influence the survival outcomes of SKCM patients, the DNA sequencing data of 1,350 RBPs and clinical information of 177 SKCM patients with RBP mutations were combined and analyzed. By taking the Kaplan–Meier test, 43 RBPs were demonstrated to carry prognosis-associated mutations (*p* < 0.05) (Supplementary Table 5). The clinical information and mutant situations of 43 RBPs are exhibited in Figure 4A. Among the 43 significant RBPs, mutations in 5 RBPs were regarded as high-risk factors (green dots), and for the rest of the 38 RBPs, the mutant type showed a lower-risk grade (yellow dots) (Figure 4B). In summary, mutations in the above 43 RBPs were found to have a significant correlation with the overall survival of SKCM patients, which indicated a promising efficiency of RBP mutations in prognosis prediction.

### Functional Enrichment Analysis and Protein–Protein Interaction (PPI) Network of ASEs

**Figure 5:**
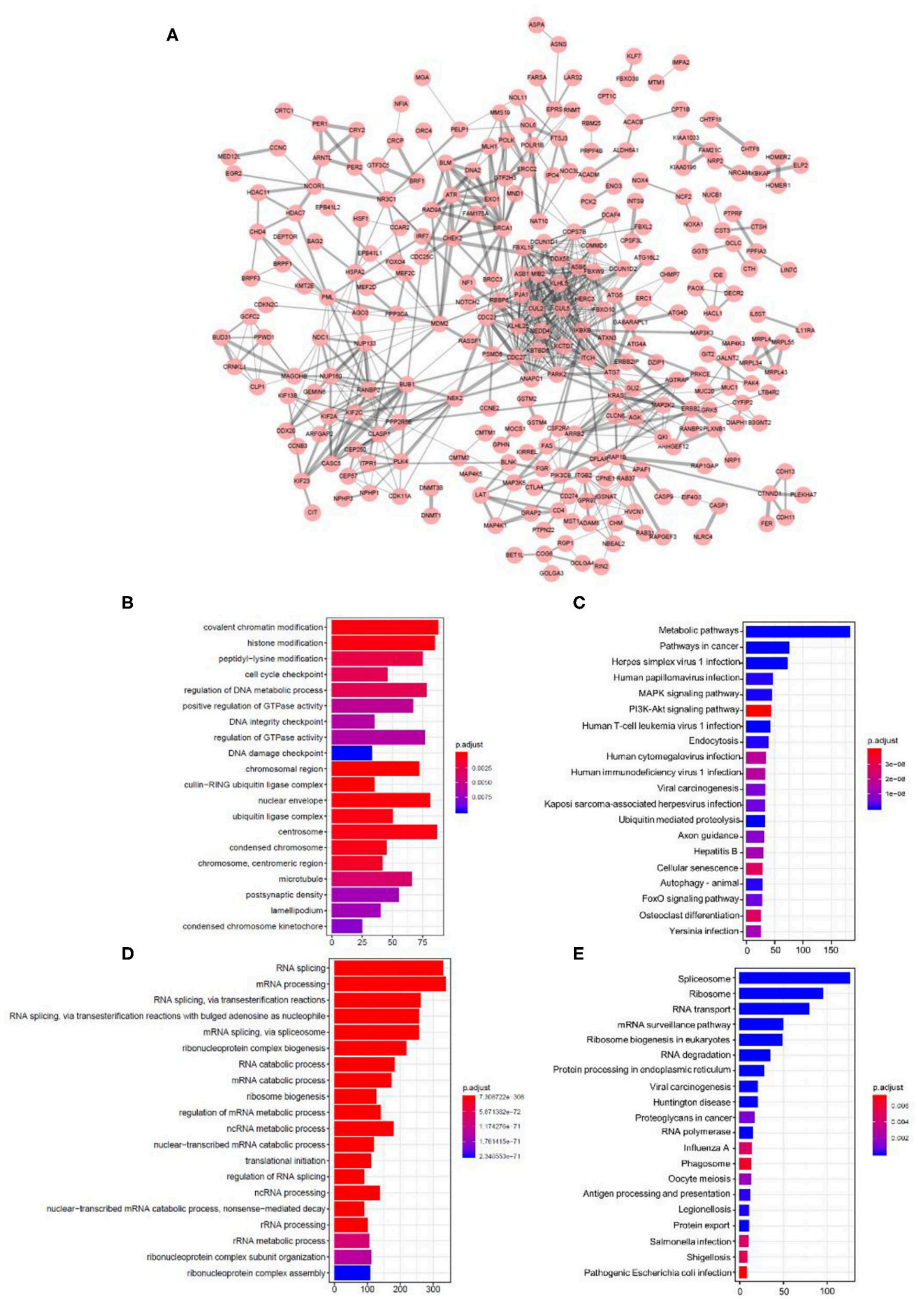
Construction of the protein–protein interaction network and functional enrichment analysis. **(A)** Protein–protein interaction network of spliced genes affected by RBP mutations. Red dots represent each spliced gene. Line thickness represents the strength of interaction. **(B–E)** GO and KEGG analysis outcomes of RBPs and spliced genes. The top 20 most significant enriched pathways were exhibited in a bar plot. **(B,C)** Enriched pathways in GO analysis, **(B)** KEGG analysis, and **(C)** spliced genes regulated by RBPs. **(D,E)** GO, **(D)** KEGG, and **(E)** enrichment analysis outcomes of RBPs.

We then tried to investigate the potential mechanism following the alterations of relevant spliced genes as well as their encoding proteins. There were 19,748 ASEs in 7,776 parent genes that showed a close correlation with RBP mutations (Supplementary Table 2, *p* < 0.01). The protein–protein interaction (PPI) network was investigated among the spliced genes of most significant ASEs (*p* < 1.00E-05). The network was built using the String database and visualized in Cytoscape (Figure 5A). To further reveal the molecular process and biological function mediated by the spliced genes, GO and KEGG enrichment analyses were applied to reveal their enriching pathways. GO analysis revealed the significant enrichment in the process of “covalent chromatin modification,” “histone modification,” “peptidyl-lysine modification,” “cell cycle checkpoint,” “DNA metabolic,” etc. (Figure 5B). As for the KEGG terms, several significant enriched pathways were closely related to tumor progress, like “metabolic pathways,” “pathways in cancer,” “MAPK signaling pathway,” and “PI3K-Akt signaling pathway” (Figure 5C). In summary, the above enrichment results revealed that these ASEs may have an essential role in the biological process and tumorigenesis of SKCM patients, by regulating the modification process, cell-cycle checkpoint, metabolic, and other cancer-related pathways.

In addition, we also made a comparison between the enrichment outcomes of RBPs and spliced genes regulated by these RBPs. The top 20 most significant enriched pathways of GO and KEGG analysis were listed. RBPs are mostly enriched in RNA splicing or other RNA-related process (Figures 5D,E), which is quite different from the spliced genes described above. The separate GO analysis results (cellular component, CC; biological process, BP; molecular function, MF) of spliced genes and RBPs were offered in Supplementary Figures 2, 3. These results indicated that RBPs and their corresponding spliced genes exhibited obvious differences in biological function and regulatory network.

### Identification of OS-ASEs and Construction of the Correlation Network

**Figure 6:**
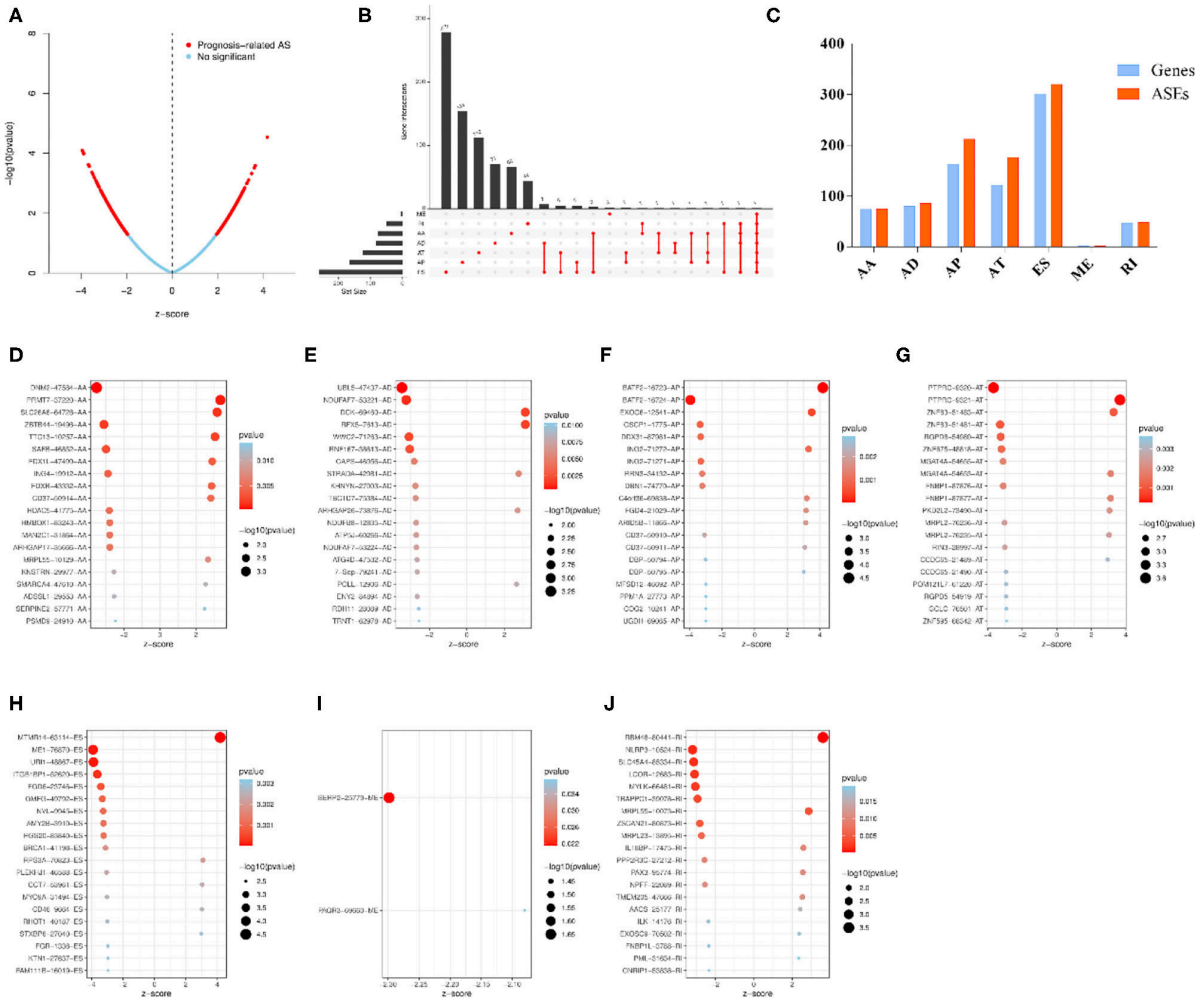
Overview of OS-ASEs affected by mRNA expression-related RBP mutations. **(A)** The volcano plot of all the ASEs. The red and blue dots represent OS-related and insignificant ASEs, respectively. **(B)** Upset plot of each type of OS-ASEs in SKCM. The black bar on the left side indicates the number of specific types of ASEs, while the red dots on the right side stand for the splicing intersections. **(C)** Distribution of seven types of ASEs and their corresponding genes. The x-axis represents each kind of ASEs (AA, AD, AP, AT, ES, RI), and the y-axis represents the number of ASEs (red) as well as the corresponding genes (blue). **(D–J)** Bubble plot of the most significant OS-ASEs in SKCM. **(D)** AA; **(E)** AD; **(F)** AP; **(G)** AT; **(H)** ES; **(I)** ME; **(J)** RI. The x-axis represents the z-score of each type of ASEs, while the y-axis stands for the OS-ASEs.

**Figure 7:**
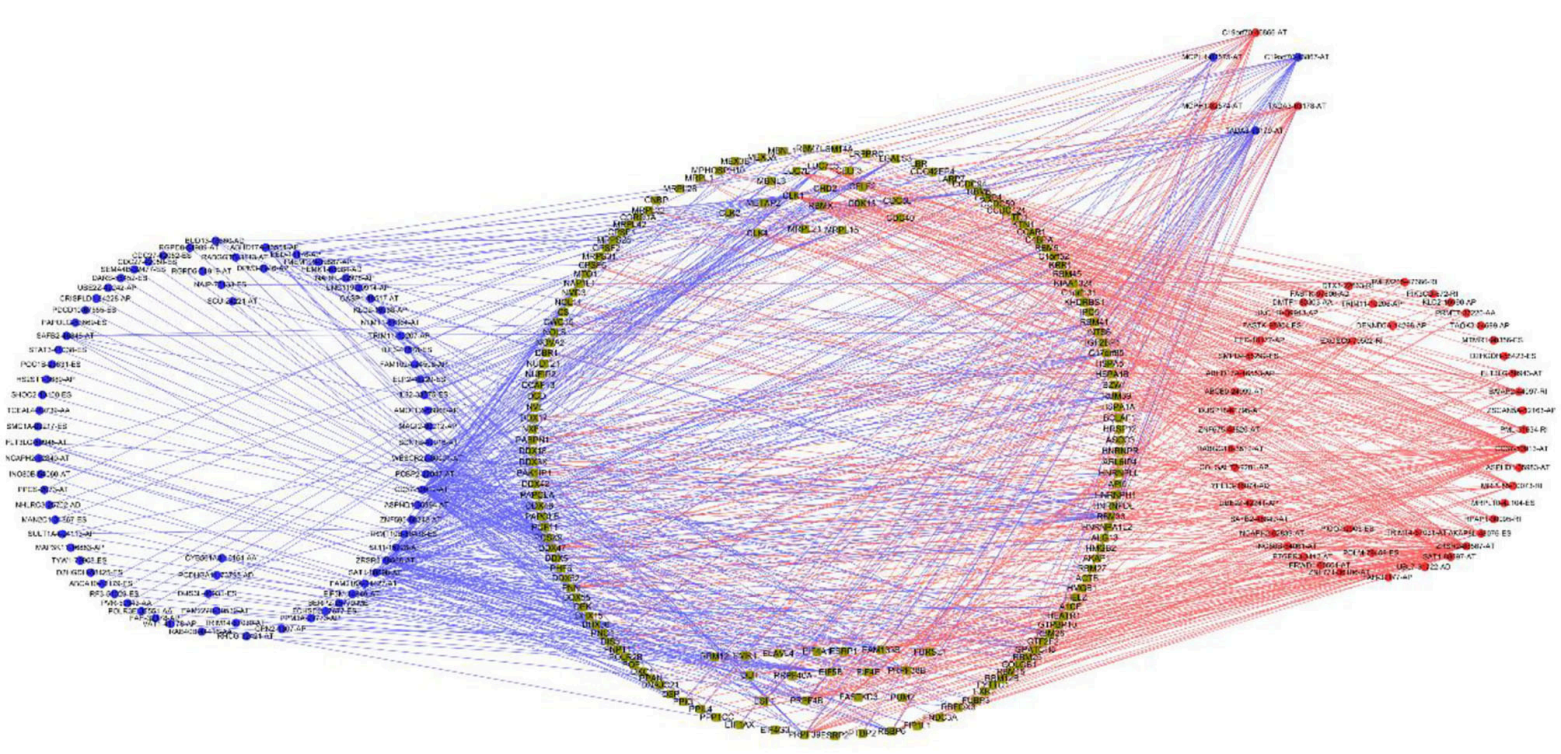
Correlation network of RBPs and OS-ASEs. The yellow dots represent RBPs. The blue and red dots indicate OS-ASEs that have been negatively regulated or positively regulated by the corresponding RBPs. The blue and red lines represent the existence of negative or positive regulation effects.

Univariable COX regression analysis was then performed to investigate all the overall survival-related ASEs (OS-ASEs) affected by RBP mutations (Supplementary Table 6). According to the volcano plot, a total of 919 OS-ASEs in 757 parent genes were identified as prognosis-associated ASEs (*p* < 0.05) (Figure 6A), which included 75 AAs in 74 genes, 86 ADs in 80 genes, 212 APs in 163 genes, 176 ATs in 122 genes, 320 ESs in 301 genes, 2 MEs in 2 genes, and 48 RIs in 47 genes (Figures 6B,C). ES was the most common splicing type associated with overall survival of SKCM patients (Figure 6C). The most significant OS-ASEs in each type of splicing patterns are shown in bubble plots (Figures 6D–J). We also constructed the splicing-regulatory network of these OS-ASEs and corresponding RBPs. The correlation network was visualized in Cytoscape. The positively regulatory (red lines, red dots) and negatively regulatory (blue lines, blue dots) relations were exhibited, respectively (Figure 7).

### Prediction Model Establishment Based on All Types of OS-ASEs

**Figure 8:**
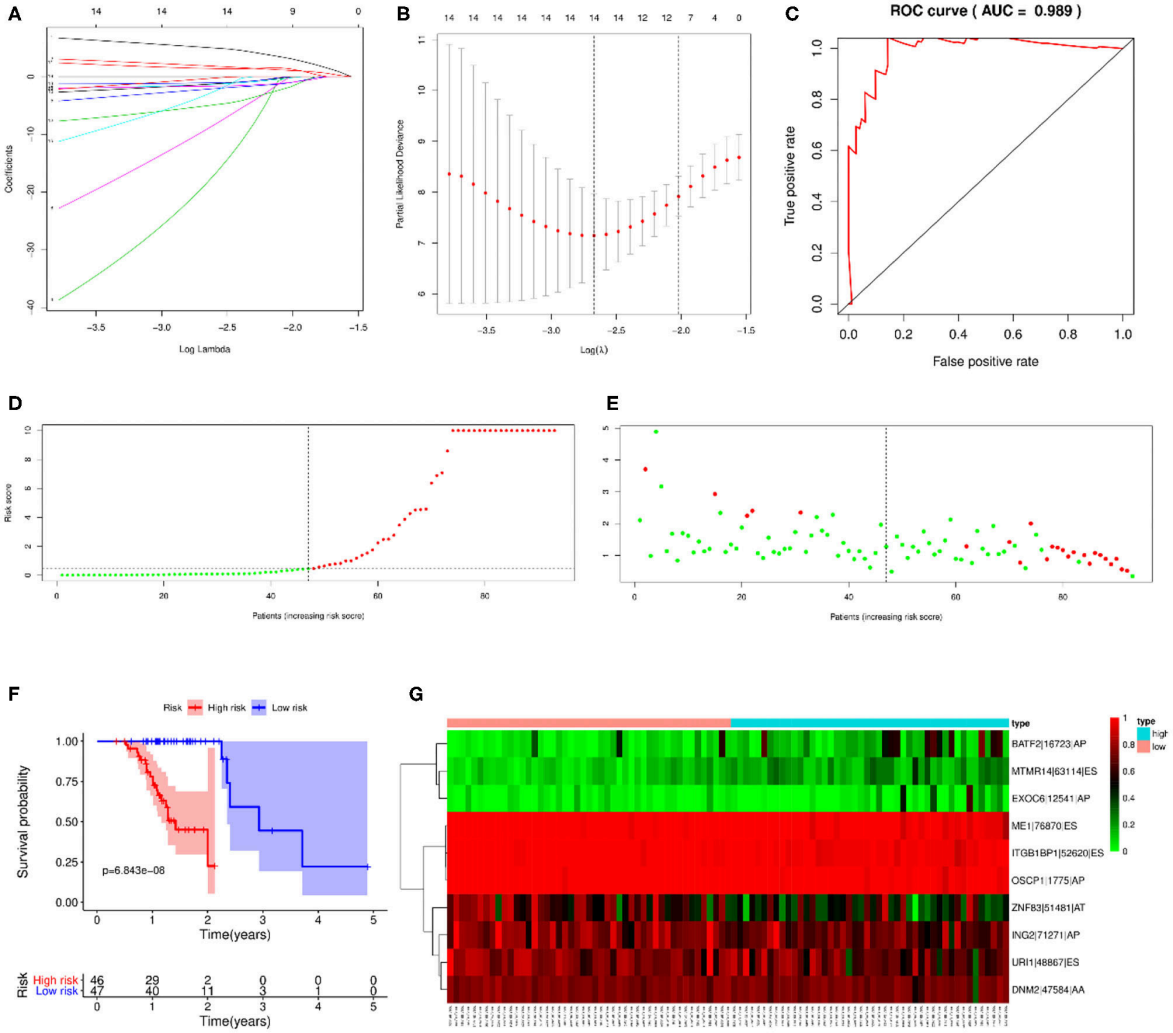
Prognostic model based on all types of OS-ASEs. **(A)** Cross-validation for tuning parameter selection in the proportional hazard model. **(B)** LASSO regression analysis for screening coefficients in all types of OS-ASEs. **(C)** The ROC curves for evaluating the efficiency of the prognostic model. **(D)** The risk curve of 93 SKCM patients matched with intact follow-up data. **(E)** The scatter plots of SKCM samples. The red and green plots represent alive and death endpoints, respectively. **(F)** Kaplan–Meier overall survival curves based on all types of OS-ASEs. The numbers of patients in the high-risk and low-risk groups at different survival times are listed at the bottom panel, respectively. **(G)** The heatmap of 10 OS-ASEs selected by LASSO regression analysis.

LASSO regression analysis and multivariate Cox regression analysis were then performed to construct a prognostic model based on all types of OS-ASEs that are influenced by RBP mutations, among which 10 OS-ASEs were selected as candidate predict factors (Figures 8A,B). As shown in Table 1, MTMR14-63114-ES, BATF2-16723-AP, and EXOC6-12541-AP were identified as a high-risk factor (HR>1), while the rest 7 ASEs indicated good prognosis (HR<1) (Table 1). The prediction efficiency was evaluated by building the ROC curve, and the AUC value was 0.989, indicating an obviously credible prediction efficiency (Figure 8C). Then, the risk score of each SKCM patient was calculated based on the prediction model and the medium cutoff value was applied to divide patients into high-risk and low-risk groups (Figures 8D,E). Kaplan–Meier survival analyses indicated that SKCM patients in the low-risk groups exhibited a significant better survival compared with those in the high-risk group (*p* = 6.843E-08) (Figure 8F). The heatmap exhibited the expression's situation of OS-ASEs in the final prognostic model (Figure 8G).

**Table 1:** Prognostic signature based on OS-ASEs.

**ID**	**Coef**	**HR**	**95% CI_L**	**95% CI_H**	***P*-value**
MTMR14|63114|ES	11.154	6.98E+04	1.25E+01	3.91E+08	1.13E-02
BATF2|16723|AP	4.800	1.22E+02	4.20E+00	3.52E+03	5.19E-03
ME1|76870|ES	−61.790	1.46E-27	8.46E-42	2.53E-13	2.21E-04
URI1|48867|ES	−8.157	2.87E-04	7.73E-07	1.06E-01	6.89E-03
ITGB1BP1|52620|ES	−56.070	4.46E-25	1.84E-38	1.08E-11	3.62E-04
DNM2|47584|AA	−7.986	3.40E-04	4.24E-07	2.73E-01	1.92E-02
EXOC6|12541|AP	5.652	2.85E+02	3.00E+00	2.71E+04	1.50E-02
OSCP1|1775|AP	−33.112	4.17E-15	3.95E-28	4.39E-02	3.04E-02
ING2|71271|AP	−3.577	2.80E-02	3.11E-04	2.52E+00	1.19E-01
ZNF83|51481|AT	−4.202	1.50E-02	1.12E-04	2.00E+00	9.25E-02

*CI, confidence interval; Coef, coefficient; HR, hazard ratio*.

We also constructed a prediction model based on RBP mutations to determine the prognosis value of RBPs themselves instead of their affections on ASEs (Supplementary Figure 4). Mutations in 13 RBPs were selected in this model (Supplementary Figures 4A,B; Supplementary Table 7), and the AUC value under the ROC curve was 0.527 (Supplementary Figure 4C). This result indicated that mutations in RBPs affect SKCM progression and outcome mostly through regulating downstream ASEs instead of themselves.

### Cox Regression Analysis of Prognosis-Related Clinical Features

**Figure 9:**
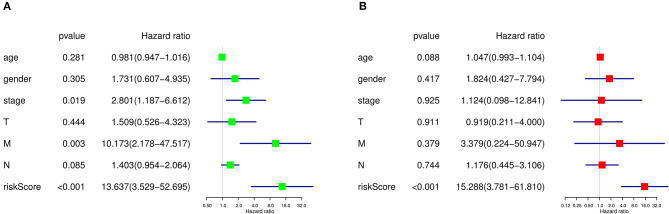
Cox regression analysis of prognosis-related clinical features and OS-ASEs**. (A)** Univariate Cox regression analysis and **(B)** multivariate Cox regression analysis.

As there were several other risk factors that may contribute to the prognosis of SKCM patients, we next performed univariate and multivariate Cox regression analyses to further evaluate the prognosis value of risk scores calculated by our prediction model and other risk factors including age, gender, tumor stage, and TMN stage. According to the univariate Cox regression analysis result, both tumor stage (HR=2.801, *P* = 0.019, 95% CI: 1.187–6.612), M stage (HR = 10.173, *P* = 0.003, 95% CI: 2.178–47.517), and risk score quartiles based on ASEs (HR=13.637, *P* = 1.517E-04, 95% CI: 3.529–52.695) are regarded as high-risk factors in terms of SKCM prognosis (Figure 9A). As for the multivariate Cox regression analysis, risk score quartiles based on ASEs is the only significant high-risk factors to SKCM patients' outcome (HR=15.288, *P* = 1.303E-04, 95% CI: 3.781–61.810) (Figure 9B). This result further corroborated that the risk score calculated by our predict model is an independent risk factor of SKCM patients' prognosis.

## Discussion

SKCM is one of the deadliest types of cancer with an increasingly annual morbidity and mortality rate (41, 42). Major etiological factors contributed to SKCM development including UV radiation, skin pigmentation reduction, nevus density increase, immunosuppression, family history, and genetic susceptibility (43, 44). Though previous studies have identified several biomarkers, efficient diagnostic and prognostic indicators are still lacking (45, 46).

In recent years, remarkable advances have been made in prognostic biomarker identification in SKCM. Except the conventional tissue-based markers such as Breslow thickness (47, 48), ulceration (47, 49, 50), mitotic rate (47, 51), tumor-infiltrating lymphocytes (52, 53), lymphatic and vascular invasion (51, 54), a great amount of research effort has been made to explore molecular biomarkers with efficient prognostic implication. Candidates including S100B (55, 56), ki-67 (57), metallothioneins (MTs) (58–60), and lactate dehydrogenase (LDH) were repeatedly demonstrated to associate with poor clinical outcomes of SKCM patients. Genetic biomarkers such as specific mutations in BRAF and NRAS were also considered to have prognostic implication in SKCM (61–63). Besides, considering the limitations in sensitivity and specificity of individual biomarkers, multi-marker arrays also gained extensive attention and several biomarker panels have been proposed (64–66). It is clear that the discovery of predict biomarkers of SKCM has been evaluated with positive results; however, challenges still remained. Conflicting results are wildly reported, and few of these biomarkers have been proven to be clinically useful or only reliable to a particular group of SKCM patients (67–70). Therefore, currently there still exists strong demand of SKCM prognostic biomarkers that provide guidance to SKCM patient management.

Almost all the multi-exon genes undergo alternative splicing (71, 72). Alternative splicing refers to a biological process that transforms a single pre-mRNA to multiple splice isoforms and finally leads to different or antagonistic functional as well as structural characteristics of protein products or results in different phenotypes due to alternating expression levels of the spliced genes (73, 74). Currently, an increasing number of evidences have been proposed to regard the alternative splicing as an effective indicator of carcinogenic processes (9, 29, 39, 75–79). Zhang et al. revealed that U2AF2 can enhance melanoma migration and cancer aggressiveness by facilitating CD44v8-10 alternative splicing, an isoform switch required for tumor prognostic and metastases (80). Besides, Liu et al. found that JMJD6 can regulate the alternative splicing of a critical component of the MAPK signal pathway, PAK1, and thereby promoting melanoma carcinogenesis (33). However, although some specific correlations between ASEs and melanoma have been identified, the exploration and prognostic signatures were developed only based on splicing events (32). The understanding and exploration of alternative splicing in SKCM are still far from enough.

ASEs were mostly regulated by a series of RBPs, whose mutations may contribute to many of AS alterations observed in cancer (39). In the past few years, increasing researches have revealed the significant role of RBP mutations in prognosis prediction in multiple cancer types (22, 39, 77, 81–83), while their role in SKCM prognosis has not been discussed yet. To determine the underlying mechanism, we deeply investigated the potential regulatory associations between RBP mutations and ASEs, so as to provide insight into the phenotype changes of ASEs in SKCM and contribute to precision treatment. We integrated the DNA-seq and RNA-seq data as well as clinical information obtained from TCGA, and ASE information from TCGA SpliceSeq. We then systematically investigated RBP mutations that correlated with mRNA expression, overall survival, and ASEs, as well as the overall situation, interaction network, and enriched pathways of related spliced genes. We also constructed predicted models based on different types of OS-ASEs, which offered us a potential intervention target for clinical SKCM treatment.

In our study, 19,748 ASEs were identified to associate with RBP mutations. Functional analysis of spliced genes revealed the enrichment in several important biological processes like modification process, cell-cycle checkpoint, DNA metabolism, MAPK signaling, and PI3K-Akt signaling. The correlation network between splicing factors and OS-ASEs was also constructed to highlight the genetic mechanism. In addition, in order to construct a prediction model and evaluate the prediction value of ASEs, LASSO regression analysis was applied to screen the prediction factors and AUC value under the ROC curves were calculated to evaluate the predict efficiency. The final prediction model based on all types of ASEs consists of 10 spliced events, including MTMR14-63114-ES, BATF2-16723-AP, ME1-76870-ES, URI1-48867-ES, ITGB1BP1-52620-ES, DNM2-47584-AA, EXOC6-12541-AP, OSCP1-1775-AP, ING2-71271-AP, and ZNF83-51481-AT. The risk scores were calculated for each SKCM patient by taking LASSO and multivariate Cox regression analysis of PSI values of OS-ASEs, and the subsequent risk stratification by risk scores enabled the satisfactory differentiation of SKCM patients with different survival outcomes. It is noteworthy that this prediction model based on all types of OS-ASEs exhibits an admirable prediction ability, with the AUC value of 0.989, which is much more significant than the previous study (32).

According to our research, we revealed a novel network between RBPs and alternative splicing changes in SKCM that provide sufficient resources of information to understand the molecular mechanism of, and potentially reverse, the tumorigenesis and survival outcomes. One core implication of our research for SKCM prognostic and clinical studies is to expand the functional effects of genetic and epigenetic alterations into changes included in the AS process of genes involved in tumor-related pathways. The prognostic model constructed in our study possessed a high performance for risk stratification in SKCM, which was promising in predicting survival outcome and helping to apply more appropriate treatment regimens to different SKCM patients. Besides, given the high prevalence of splicing defects in tumor, several small molecule modulators targeting RNA processing have been explored and exhibited promising therapeutic effects in tumor treatment (84). Our study also provides potential targets for SKCM treatment. Certainly, more researches concerning the molecular mechanism of significant RBP mutations on AS regulation are needed in the future. Functional investigations and biological experiments focused on the significance of ASEs, and how these aberrant procedures affect critical cancer pathways may provide novel therapeutic targets for SKCM treatment and improve survival outcomes.

In conclusion, our research described a comprehensive landscape of aberrant alternative splicing and its regulation in SKCM. We focused on the executors of splicing procedure, as well as systematic analysis mutation and gene expression patterns of 1,350 RBP genes. We also constructed a network to exhibit the potential correlation between RBPs and relevant ASEs. Furthermore, we identify enrichment of tumor-critical pathways among the spliced genes. The prediction model constructed in this study enabled the satisfactory differentiation of overall survival and provided a better guidance for clinical decisions and prognosis prediction of SKCM patients.

## Data Availability Statement

The datasets generated for this study can be found in the TCGA and TCGA SpliceSeq.

## Author Contributions

Conceptualization: CM, P-YS, and Z-QL. Methodology: CM, P-YS, and XL. Investigation: CM and P-YS. Writing—original draft: CM. Writing—review and editing: WZ and H-HZ. Funding acquisition: Z-QL. Resources: CM, P-YS, and XL. Supervision: Z-QL. All authors contributed to the article and approved the submitted version.

## Conflict of Interest

The authors declare that the research was conducted in the absence of any commercial or financial relationships that could be construed as a potential conflict of interest.

## Supplementary Material

Supplementary Figure 1 Waterfall plot of 91 RBPs with mRNA expression-affected mutations.

Supplementary Figure 2 GO analysis of spliced genes regulated by RBP mutations**. (A)** Cellular component; **(B)** biological process; **(C)** molecular function. The x-axis stands for the gene ratio (the proportion of genes participated in each pathway) and the y-axis represents the name of pathways.

Supplementary Figure 3 GO analysis of 1350 RBPs. **(A)** Cellular component; **(B)** biological process; **(C)** molecular function. The x-axis stands for the gene ratio and the y-axis indicates the pathway names.

Supplementary Figure 4 Prognostic model based on 1350 RBPs. **(A)** LASSO regression path plot for building predict model and calculating coefficients; **(B)** LASSO coefficient profiles based on all types of OS-ASEs; **(C)** construction of ROC curves to evaluate the predict efficiency.

Supplementary Table 1 Gene list of 1350 known and predict RNA binding proteins (RBPs).

Supplementary Table 2 RBPs mutations induced splicing alterations. The left table lists all the splicing events affect by RBP mutations (Splicing_event) as well as their corresponding RBPs (RBP). It also provides the corresponding *p*-value (*p*-value) of each splicing event. The right table lists the occurrence frequency of different splicing patterns and their parent genes.

Supplementary Table 3 Information of 91 RBPs selected by Wilcoxon Mann-Whitney test. The table includes the list of 91 RBPs (RBP), corresponding p-value (p.value) and the percentage of mutant samples (Mut_ratio). It also provides the sample size (Mut_count/Wild_count) and the median of mRNA expression (Wild_med/Mut_med) of wild and mutant samples, respectively.

Supplementary Table 4 Corresponding splicing events regulated by 91 RBPs. In the left table, a total of 1,728 splicing events in 1,268 parent genes were identified using Pearson correlation test. The information of each RBPs and their related splicing events were listed, and the Pearson correlation coefficient (Cor) and p.value were also provided in this table. “Positive” and “negative” in column Reg. indicated “positive regulation and “negative regulation,” respectively. The occurrence frequency of each types of splicing event and their parent genes were listed in the right table.

Supplementary Table 5 Kaplan-Meier test result of 45 significant RBPs. RBP mutations that potentially affect the survival outcomes of SKCM patients were selected by Kaplan-Meier test. The *p*-value, hazard ratio (HR), 95% confidence interval (95% CI) of each RBP were listed.

Supplementary Table 6 Survival-related splicing events selected by Lasso regression. Among the 19748 splicing events affected by RBP mutations, a total of 919 splicing events were identified to be correlated with the overall survival of SKCM patients using Lasso regression. The Z_score (Cor), hazard ratio (HR), 95% confidence interval (95%CI_L and 95%CI_H, respectively) and *p*-value were listed in the left table. The occurrence frequency of each types of splicing event and their parent genes were listed in the right table.

Supplementary Table 7 Construction of predict model based on RBP mutations. RBP mutations were selected by Lasso regression. A total of 13 RBPs were selected into the predict model using multivariate Cox regression analysis. The coefficient (Coef), hazard ratio (HR), 95% confidence interval (95%CI_L and 95%CI_H, respectively) and *p*-value were listed.
