# Supplementary figures and images for: Aberrant RNA Splicing Events Driven by Mutations of RNA-Binding Proteins as Indicators for Skin Cutaneous Melanoma Prognosis
Source: Front Oncol. 2020 Oct 15;10:568469. doi: 10.3389/fonc.2020.568469 (PMC7593665; doi:10.3389/fonc.2020.568469)

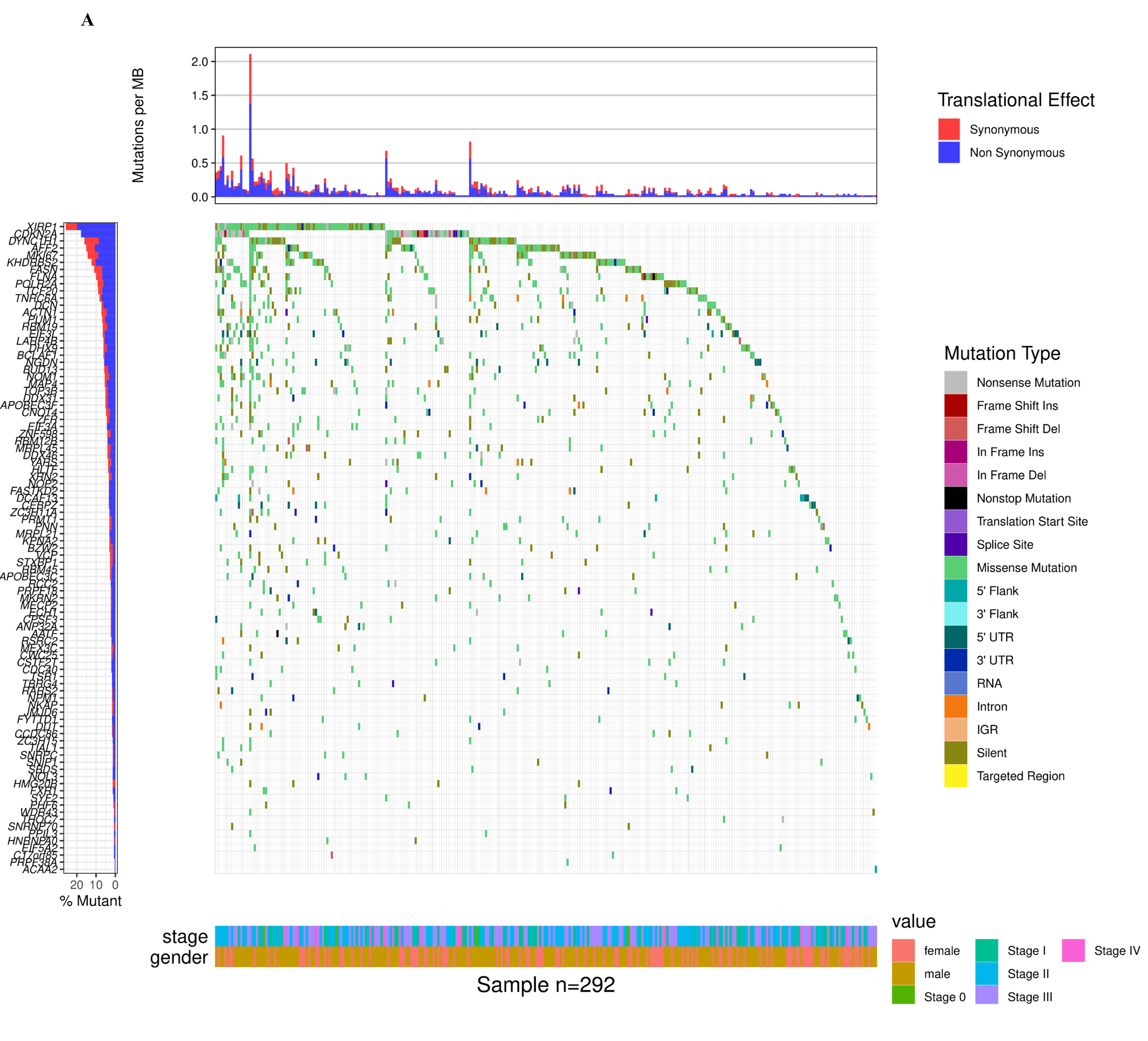

Supplement: Supplementary Figure 1 — Waterfall plot of 91 RBPs with mRNA expression-affected mutations. [file Image_1.TIF]

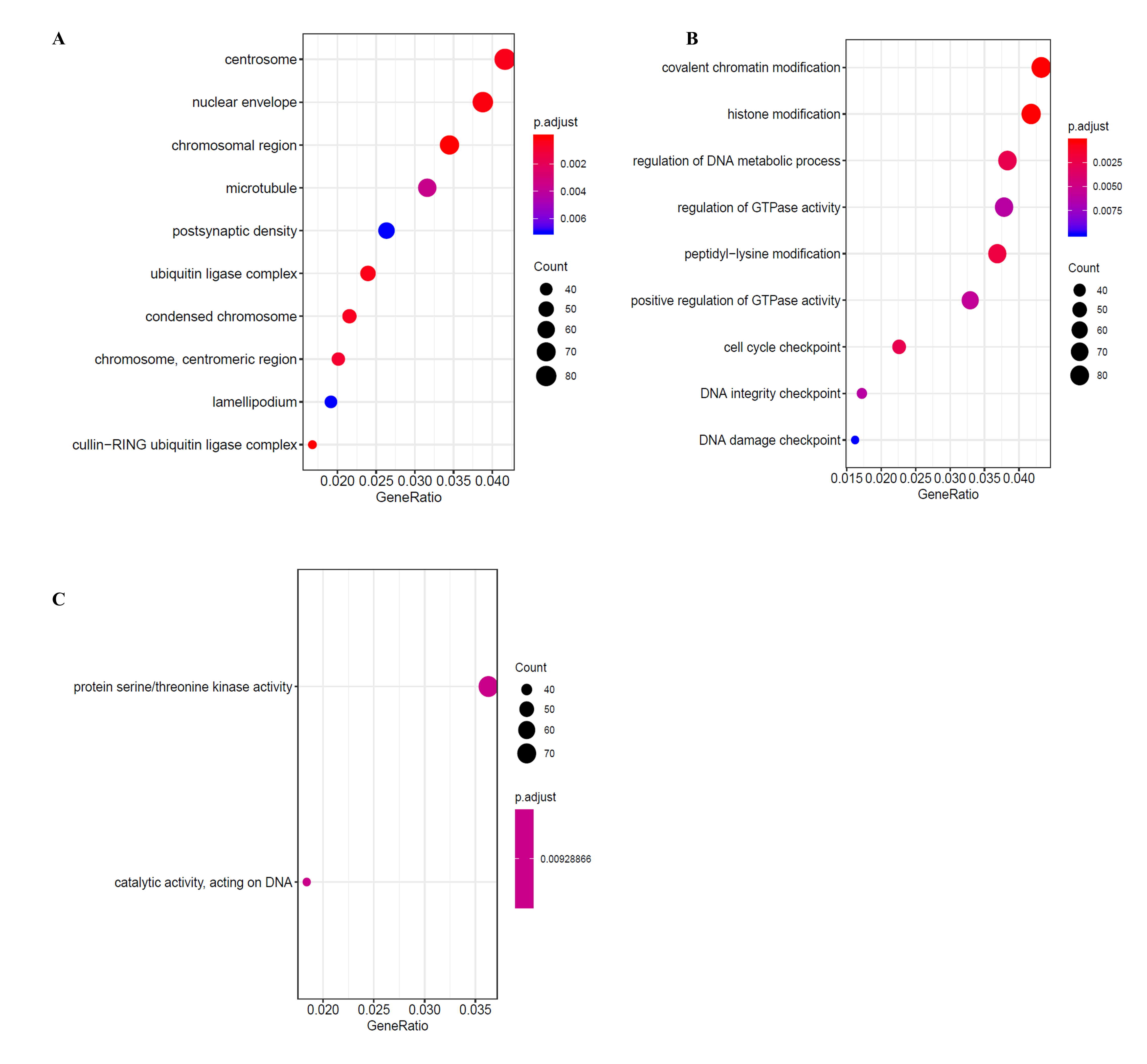

Supplement: Supplementary Figure 2 — GO analysis of spliced genes regulated by RBP mutations. (A) Cellular component; (B) biological process; (C) molecular function. The x-axis stands for the gene ratio (the proportion of genes participated in each pathway) and the y-axis represents the name of pathways. [file Image_2.TIF]

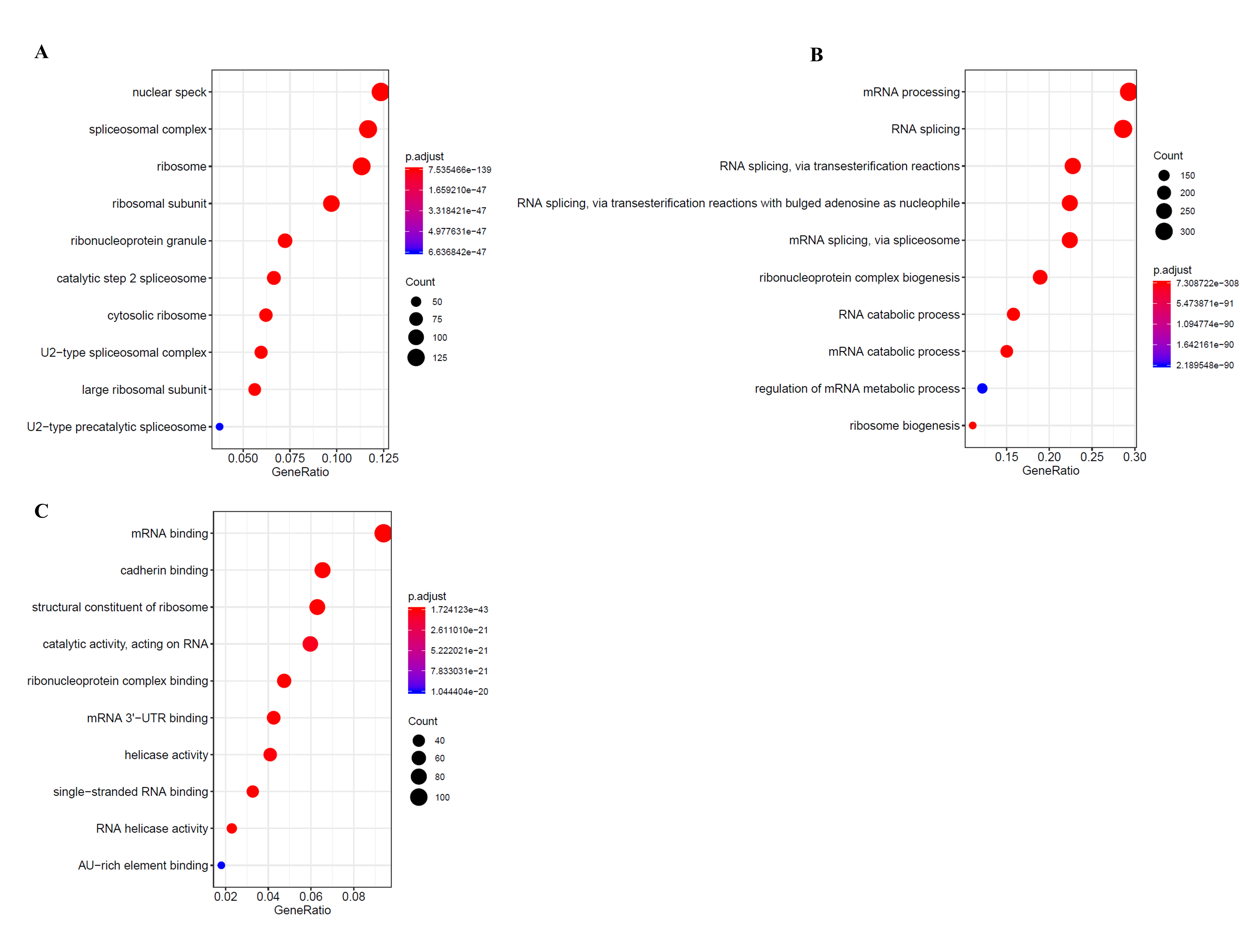

Supplement: Supplementary Figure 3 — GO analysis of 1350 RBPs. (A) Cellular component; (B) biological process; (C) molecular function. The x-axis stands for the gene ratio and the y-axis indicates the pathway names. [file Image_3.TIF]

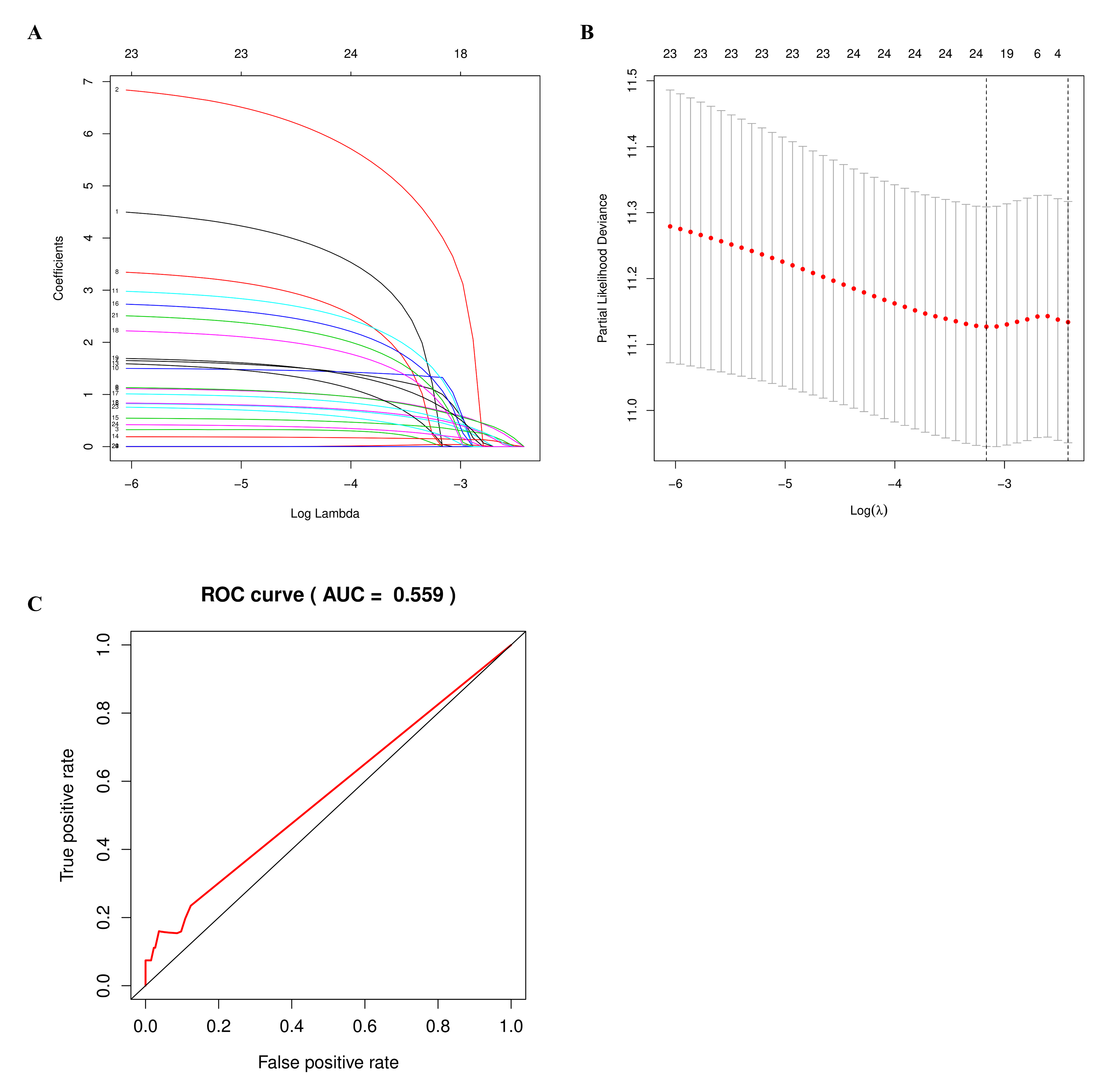

Supplement: Supplementary Figure 4 — Prognostic model based on 1350 RBPs. (A) LASSO regression path plot for building predict model and calculating coefficients; (B) LASSO coefficient profiles based on all types of OS-ASEs; (C) construction of ROC curves to evaluate the predict efficiency. [file Image_4.TIF]
